# Rectus Abdominis Muscle Malignant Fibrous Histiocytoma Causing a Large Abdominal Wall Defect: Reconstruction with Biological Mesh

**DOI:** 10.1155/2014/723851

**Published:** 2014-02-06

**Authors:** Evangelos Falidas, Stavros Gourgiotis, Christina Goudeli, Stavros Mathioulakis, Konstantinos Vlachos, Constantinos Villias

**Affiliations:** First Surgical Department, 417 NIMTS Military Veterans' Fund Hospital of Athens, 12 Monis Petraki, 11521 Athens, Greece

## Abstract

Malignant fibrous histiocytoma (MFH) is a common soft tissue sarcoma usually involving limbs and retroperitoneum. MFH of the rectus abdominis muscle is extremely rare. Surgery in similar cases leads to large abdominal wall defects needing reconstruction. Biological and synthetic laminar absorbable prostheses are available for the repair of hernia defects in the abdominal wall. They share the important feature of being gradually degraded in the host, resulting the formation of a neotissue. We herein report the case of an 84-year-old man with MFH of the rectus abdominis muscle which was resected and the large abdominal wall defect was successfully repaired with a biological mesh.

## 1. Introduction

Malignant fibrous histiocytoma (MFH) is a pleomorphic sarcoma. It was first described as malignant histiocytoma and fibrous xanthoma by Ozzello et al. [1] and was established a soft tissue sarcoma arising from fibroblasts and histiocytes [1, 2]. MFH is a relatively rare tumor that occurs throughout the body [3]. However, it is also the most common sarcoma appearing during the 6th and 7th decades, while men are more often affected than women. The most frequent site of MFH is the extremities (lower extremity 49%, upper extremity 19%) followed by the retroperitoneum (16%) and peritoneal cavity (5%–10%) [4].

The introduction of biological meshes (BMs) like Permacol (PM), Strattice, and Surgisis has opened new alternatives. BMs products have several potential advantages over other synthetic permanent materials in selected clinical situations. Indications for implantation of a BM in abdominal wall reconstruction include contaminated wounds, complex repairs at high risk for developing wound-healing problems, high likelihood of a cutaneous exposure, and unavoidable direct placement of mesh over bowel [5].

We herein report the case of an 84-year-old man with a MFH in the rectus abdominis muscle, treated by removal of the tumor and the muscle, which was reconstructed with BM.

## 2. Case Presentation

An 84-year-old male was admitted complaining about abdominal pain and a palpable mass in the abdominal wall. His medical history included atrial fibrillation, chronic obstructive pulmonary disease, and open cholecystectomy performed 2 years ago. During the clinical examination, a large, immobile, and slightly painful mass was palpated at the level of the left rectus abdominis muscle. Abnormal laboratory findings included leukocytosis (14,323/mm³), Hb: 9 g/dL, and INR: 4.

**Figure 1:**
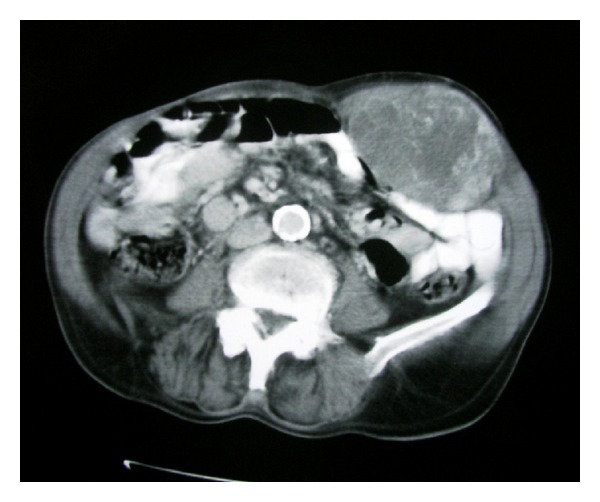
The abdominal CT demonstrates a 11 × 7 cm mass in diameter arisen from the rectus abdominis muscle.

Although the patient's reported history of a two-month growing mass, spontaneous rectus abdominis rupture was initially suspected. The abdominal ultrasound (U/S) demonstrated a hyperechogenic mass limited to the abdominal wall. The abdominal computed tomography (CT) described a large mass of 11 × 7 cm in diameter, arising from the rectus abdominis muscle with sarcomatous characteristics (Figure 1). No signs of liver, lungs, and nodal involvement were demonstrated.

**Figure 2:**
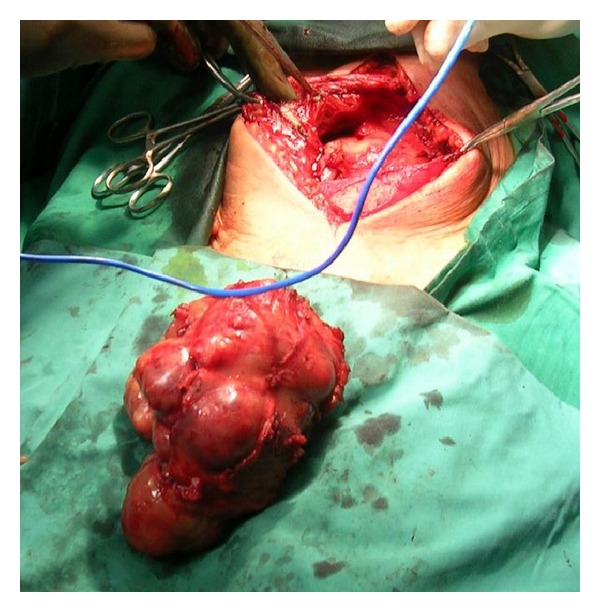
The en bloc resection of the tumor, the rectus abdominis, and the muscular sheath.

**Figure 3:**
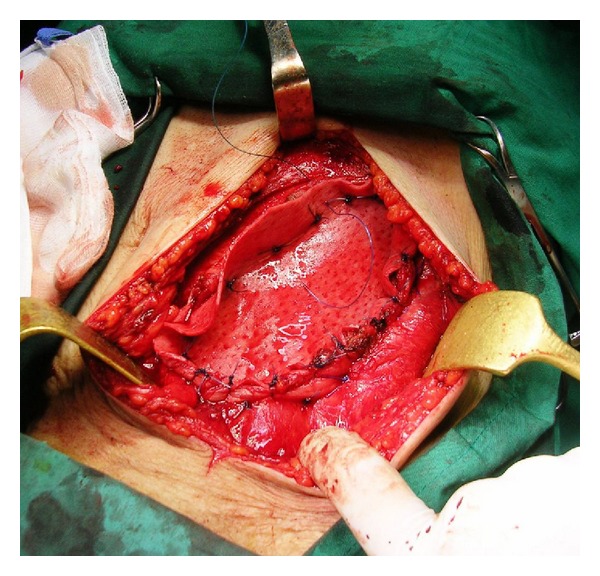
The biological mesh was directly posted over the intestinal loops; stay sutures were posted on the left ipsilateral internal oblique muscle and the posterior sheath-linea alba of the contralateral rectus abdominis muscle.

Anemia and coagulation parameters were adjusted and the patient underwent surgery. Through a paramedian incision, en bloc resection of the tumor, the rectus abdominis, and the muscular sheath was performed (Figure 2). Abdominal defect was repaired using porcine acellular mesh. The mesh was directly posted over the intestinal loops while stay sutures were posted on the left ipsilateral internal oblique muscle and the posterior sheath-linea alba of the contralateral rectus abdominis muscle (Figure 3). The external oblique muscle, the subcutaneous tissue, and the skin were closed over the mesh. Two suction drains were placed laterally between the mesh and the subcutaneous tissue, and the skin was closed with a nylon suture. The patient's postoperative course was uneventful. Pathology report described MFH. Although the patient refused further treatment, no tumor and hernia signs were observed 12 months later.

## 3. Discussion

MFH generally refers to the group of tumors that originate from histiocytes and make their specific diagnosis difficult since these lesions have a variety of pathological types [6]. They usually consist of 3 types of cells: spindle shaped, round, and giant cells. The pathologic findings greatly vary and it can be classified into 5 types depending on the distribution of these cells: the storiform-pleomorphic, the myxoid, the giant cell, the inflammatory cell, and the angiomatoid type. The storiform-pleomorphic type is the most common and it accounts for approximately 65% of MFH [7].

The most common symptoms are abdominal pain, fatigue, weight loss, and a palpable mass. Specifically, in the cases of abdominal and retroperitoneal MFH, hematuria, lower extremity pain, abdominal distension, varicose veins, and hernia could be observed [6]. MFHs have a high likelihood of metastasis and recurrence. The patients with occurrence in the retroperitoneal cavity sometimes do not have any symptoms and these tumors are often found in an advanced stage and especially for the rare cases where this tumor occurs in psoas muscle.

The main treatment is extensive surgical resection in comparison with concurrent chemotherapy and radiation therapy to reduce the possibility of local recurrence and metastasis. It has been reported that the average 5-year survival rate of patients with MFH is 59% to 66.7% and the local recurrence rate is 16% to 31% [7]. The prognostic factors to predict local recurrence and distant metastasis include the patient's age, the history of a recurrence, the tumor size, the depth of invasion, the histological grade of the mass, and the status of the resection tumor margin [7].

Radical treatment often results in extended abdominal wall defects that need reconstruction. Various options of abdominal wall reconstruction exist. Primary closure does not always respect the principles of tension-free repair while it often results in an incisional hernia. Plastic surgery techniques such as pedicled or free myocutaneous flaps need advanced surgical skills and are time-consuming procedures that limit a wide application especially in patients with comorbidities.

On the other hand, surgery with permanent synthetic meshes (such as polyester, polypropylene, and expanded polytetrafluoroethylene) despite providing satisfactory results is often complicated by foreign body reaction, erosion of adjacent viscera, migration, intestinal obstruction, or fistula formation. BM structure is based on acellular porcine collagen or human cadaveric collagen. These meshes are not allergenic, incorporate easily into host tissue, do not cause a foreign body reaction, and present a rapid colonization of host tissue cells and blood vessels increasing resistance to infection. They also present a high tensile strength being at the same time soft and flexible. Less adhesion formation is a grade advantage considering the fact that it can be applied directly in contact with the abdominal viscera [8]. Two types of materials exist: those with cross-links that stabilize the collagen molecule, thus preventing its rapid degradation, and those noncross-linked, which undergo a progressive and variable degradation over time [9]. Clearly, the process for which these prostheses are designed is not feasible for the majority of the synthetic polymeric prostheses, which remain for life in the recipient organism; in certain instances, these synthetic prostheses elicit inflammatory and foreign body reactions with the potential for more diverse postimplant complications [10].

The advantage of using BMs is that the repair mechanisms approach optimal conditions. However, there may also be inconveniences, including adverse effects that have been described after implantation [11]. One of the areas for improvement and research is the control of the prosthetic degradation times, particularly of noncross-linked prostheses.

In the reported case, the abdominal defect was repaired using porcine acellular mesh. The mesh was directly posted over the intestinal loops while the external oblique muscle, the subcutaneous tissue, and the skin were closed over the mesh. Two suction drains were placed laterally between the mesh and the subcutaneous tissue while the patient's postoperative course was uneventful.

## 4. Conclusion

MFH of the rectus abdominis is a rare but also existing abdominal wall tumor. BMs seem to offer a valid and rapid surgical option in similar cases where age and comorbidities limit complex reconstructions of the abdominal wall and where resections should always maintain a radical oncologic character. BM is an option for use in early closure of abdominal wall defects in potentially contaminated wounds, even when skin cover is not attainable at first, hence leaving the graft exposed. It allows earlier closure of the abdomen as well as earlier discharge of patients.
